# Encapsulation and Characterization of Gentamicin Sulfate in the Collagen Added Electrospun Nanofibers for Skin Regeneration

**DOI:** 10.3390/jfb9020036

**Published:** 2018-05-18

**Authors:** Wan Khartini Wan Abdul Khodir, Abdul Hakim Abdul Razak, Min Hwei Ng, Vincenzo Guarino, Deny Susanti

**Affiliations:** 1 Department of Chemistry, Kulliyyah of Science, International Islamic University of Malaysia Kuantan Campus, Bandar Indera Mahkota, Kuantan 25200, Pahang, Malaysia; akimedentorai@gmail.com (A.H.A.R.); deny@iium.edu.my (D.S.); 2 Tissue Engineering Centre, Universiti Kebangsaan Malaysia Medical Centre, Kuala Lumpur 56000, Malaysia; angelaster3@gmail.com; 3 Institute for Polymers, Composites and Biomaterials, National Research Council of Italy, Mostra d’Oltremare, Pad. 20, V. le Kennedy 54, 80125 Naples, Italy

**Keywords:** collagen, gentamicin, nanofibers, controlled release, skin regeneration

## Abstract

In the current practice, the clinical use of conventional skin substitutes such as autogenous skin grafts have shown several problems, mainly with respect to limited sources and donor site morbidity. In order to overcome these limitations, the use of smart synthetic biomaterials is tremendously diffusing as skin substitutes. Indeed, engineered skin grafts or analogues frequently play an important role in the treatment of chronic skin wounds, by supporting the regeneration of newly formed tissue, and at the same time preventing infections during the long-term treatment. In this context, natural proteins such as collagen—natively present in the skin tissue—embedded in synthetic polymers (i.e., PCL) allow the development of micro-structured matrices able to mimic the functions and to structure of the surrounding extracellular matrix. Moreover, the encapsulation of drugs, such as gentamicin sulfate, also improves the bioactivity of nanofibers, due to the efficient loading and a controlled drug release towards the site of interest. Herein, we have done a preliminary investigation on the capability of gentamicin sulfate, loaded into collagen-added nanofibers, for the controlled release in local infection treatments. Experimental studies have demonstrated that collagen added fibers can be efficaciously used to administrate gentamicin for 72 h without any toxic in vitro response, thus emerging as a valid candidate for the therapeutic treatment of infected wounds.

## 1. Introduction

In dermal tissue, an extracellular matrix (ECM) is a collection of extracellular molecules secreted by cells that provides spatial and mechanical signals to cells and physical support to tissues. ECM is composed mainly of collagen, elastin, and reticular fibers [1,2,3]. Therefore, it is mandatory to design skin substitutes that can mimic the dermal ECM of damaged tissue or organ to sequentially regenerate [4,5,6]. In this context, nanofiber scaffold may efficaciously provide a three-dimensional (3D) template for cell migration, attachment, and proliferation, thus supporting the healing mechanisms [7,8,9]. Electrospinning is a common fabrication technique where it is extensively used in tissue engineering applications because of its ease of fabrication and its resemblance to nano-topographical elements in the extracellular matrix of tissues [10,11,12,13,14,15].

First of all, a high surface area to volume ratio of electrospun nanofibers allows greater epithelial and endothelial cell attachment, growth, and cellular differentiation and better contour to injuries with a higher surface area for wound contact [4,16]. The main advantages of using synthetic nanofibers such as polycaprolactone (PCL) are the elimination of contamination and disease transmission and increased manufacturing uniformity [2]. However, biopolymers from a natural source (i.e., proteins, polysaccharides) are still the best choice in terms of biological recognition, and are the best in reproducing chemical signals exerted to cells by local microenvironment. Collagen has been variously used in combination with biodegradable polymers such as polycaprolactone (PCL) or polylactid acid (PLA) in order to combine their recognized in vivo biochemical/biological stability, to the higher biomechanical properties of synthetic phases [17,18,19]. 

In particular, PCL is a semi-crystalline and hydrophobic polymer approved by FDA and is frequently used in tissue engineering field [10], due to optimal mechanical properties coupled with long degradation times in vivo [20]. The addition of collagen to PCL to form scaffolds in different forms (i.e., sponges [21], biodegradable films [22], and nanofibers [23]) has in fact improved interaction with cells, promotion of adhesion and/or differentiation mechanism for the recruitment of a newly formed ECM matter, for in vitro and in vivo regeneration [4,19,20,24]. Indeed, hydrophilic natural proteins offer specific binding sites for cell adhesion while synthetic polymers confer to the scaffold a more efficient mechanical support [25]. In this case, there are a few weaknesses in terms of biocompatibility which is, mainly due to residual traces of aggressive organic solvents such as fluorinate (i.e., trifluoroethanol (TFE), hexafluoropropanol (HFIP) [26,27]) used during the fiber making. Accordingly, it has been recently discovered that HFIP may influence tertiary and quaternary structural organization of protein sequences, thus irreversibly converting collagen to gelatin macromolecules [28]. Hence, it is mandatory to identify polymer/solvent mixtures which assure high workability and chemical stability (i.e., no denaturation), preserving the biological response.

Recently, a growing interest has emerged for the optimization of fiber processing to encapsulate drug molecules in order to improve molecular transport mechanisms, thus enhancing the biological activity of the scaffold [15,29]. Previous works have confirmed that peculiar morphological properties of electrospun fibers a (i.e., high surface volume ratio) improve spatial homogeneity and encapsulation efficiency of the drug molecules [30] with respect to conventional casted films. Moreover, they offer the possibilities to easily create temporal/spatial molecular gradients which is suitable to trigger specific functionalities of the complex tissue microenvironment during regeneration [31]. More interestingly, they may enable fabrication of locally controlled release systems suitable as alternative strategy to conventional dosage forms for improved therapeutic effects, reduced frequency of drug intake, and lower level of toxicity [9].

In this work, PCL/collagen electrospun fibers (CP) is proposed to be used for the in vitro delivery of gentamicin—an antibiotic in order to prevent or, at least, to fight wound infection—thus promoting healing mechanisms. Gentamicin, a highly water-soluble drug, is an aminoglycoside molecule that exhibits bactericidal activity against a broad spectrum of microorganisms, such as *Pseudomonas aeruginosa*, *Escherichia coli*, and *Staphylococcus aureus* [31,32]. The aim of this study is to validate the potential use of gentamicin-loaded collagen/PCL nanofibers (GCP) and fabricated via electrospinning technique for skin tissue engineering. For this purpose, a preliminary study is reported, including the evaluation of fiber morphology, drug release, and in vitro cytotoxicity.

## 2. Materials and Methods

### 2.1. Materials

Polycaprolactone (PCL; Mw = 80,000) was purchased from Sigma-Aldrich (Gillingham, UK). Collagen from bovine Achilles tendon was supplied by Tissue Engineering Centre, Hospital Universiti Kebangsaan Malaysia Medical Centre. Gentamicin sulphate was obtained from Henan Province, China. Chloroform and methanol were purchased from Merck (Darmstadt, Germany).

### 2.2. Preparation of Electrospun Fibres

Collagen and PCL solution were separately dissolved in a solvent mixture (10% *w/v*) with a volume ratio of 3:1 chloroform/methanol (*v/v*) by gently stirred at room temperature for 24 h as suggested by Gautam et al. [30]. Then the, collagen and PCL were mixed at a ratio: 1:3 (*v/v*) and were kept stirred to form a homogenous solution for 24 h. For GCP, a 3% *w/v* gentamicin sulfate was added to the polymer solution and was stirred for 3 h to obtain a homogeneous solution as well. CP and GCP nanofibers were fabricated by using electrospinning homemade machine. A high voltage (Gamma High Voltage, Ormond Beach, FL, USA) was used at 16 kV and a constant flow rate (KD scientific, Holliston, MA, USA) was fixed at 0.6 mL/h. The distance between the ground collector and the needle (22 G) was fixed to 12 cm. All samples were prepared and electrospun under ambient conditions of 28 °C temperature with 50% ± 5% relative humidity. The samples were dried at room temperature to remove any residual solvents.

### 2.3. Morphological Characterization

Morphology of CP and GCP nanofibers were examined by scanning electron microscopy (EVO^®^ 50 series, Carl Zeiss AG, Oberkochen, Germany) with accelerating voltage of 5 kV under high vacuum condition. Moreover, samples were previously sputtered with a gold–palladium mixture for 3 min under vacuum. Average fiber diameter distributions were estimated using Image J^®^ software (version 3.7) (National Institute of Mental Health, Bethesda, MD, USA) on five random images at the same magnification with scale bar 1 µm (ca. 20 fibers for each image).

### 2.4. In Vitro Drug Release

The drug release profile was determined by soaking the nanofibers in triplicate in distilled water. Samples were cut into rectangles (2 cm × 2 cm), weighed and incubated in 20 mL distilled water at 37 °C.

The solutions were collected for about 24 h, in order to obtain samples of about 50 microns in thickness and a bioactive molecules density (collagen plus gentamicin) equal to 0.027 g/cm^2^. At pre-determined time intervals, 4 mL of soaking solution was collected and analyzed using UV–vis spectrophotometry Lambda 35 (Perkin Elmer, Waltham, MA, USA) at 201 nm. The solutions were returned back to the flask to maintain the sink volume. The calibration curve (Y= 0.3585X − 0.0011; r = 0.9915) constructed from a series of gentamicin solutions (0.3, 0.6, 1.25, 2.5, 5 mg/mL) as a standard concentration. The cumulative drug release was calculated using the following equation

$$\frac{\mathrm{Total}\;\mathrm{amount}\;\mathrm{drug}\;\mathrm{released}\;\mathrm{from}\;\mathrm{the}\;\mathrm{membrane}}{\mathrm{Total}\;\mathrm{amount}\;\mathrm{of}\;\mathrm{drug}\;\mathrm{present}\;\mathrm{in}\;\mathrm{the}\;\mathrm{membrane}\;}\times 100\%$$

The encapsulation efficiency was calculated by the following equation

$$\frac{\mathrm{Weight}\;\mathrm{of}\;\mathrm{drug}\;\mathrm{in}\;\mathrm{the}\;\mathrm{nanofibers}}{\mathrm{Theoretical}\;\mathrm{weight}\;\mathrm{of}\;\mathrm{drug}\;\mathrm{in}\;\mathrm{the}\;\mathrm{nanofibers}}\times 100\%$$

Therefore, a graph was plotted to determine the cumulative release of gentamicin (%) vs. time (hours).

### 2.5. Cell Culture

Biological assays were performed using a human dermal fibroblast (HDF) stem cell line (PCS-201-012) purchased from American Type Culture Collection (ATCC), Manassas, VA, USA. HDF stem cells were cultured in 75-cm^2^ cell culture flasks in Dulbecco’s modified Eagle medium (DMEM) supplemented with 10% fetal bovine serum, antibiotic solution (streptomycin 100 μg/mL and penicillin 100 μg/mL) at a density of 1.0–2.0 × 10^5^ cells/mL and had been cultures in each well. Cells were incubated under standard culture conditions (37 °C, 5% CO_2_, and 95% air) and the culture medium was replenished every day.

### 2.6. In Vitro Cytotoxicity Test

Cytotoxicity responses of CP and GCP nanofibers to HDF cells were determined by using 3-(4,5-dimethylthiazol-2-yl)-2,5-diphenyltetrazolium bromide (MTT) assay (Gibco, Invitrogen Corporation, Waltham, MA, USA). CP and GCP nanofibers were put onto 24-well plates in triplicate and incubated after adding 1 mL of α-MEM basal complete media to each well. After 1, 3, and 5 days, the HDF cells were washed with phosphate buffer solution (PBS) and media were removed and replenished with 20 μL of MTT solution (5 mg/mL) and the plates were further incubated at 37 °C, with 5% CO_2_ and 95% air humidity for 4 h. The supernatant was removed and 100 μL dimethyl sulfoxide (DMSO) was added to stop the MTT reaction and soluble the formazan crystal. Then supernatants were then transferred into a 96-well plate for optical density (O.D.) measurements at 570 nm with a plate reader. The determination of cell toxicity of the materials was presented as percentage of cell viability when compared to control cell cultured in medium only (K).

### 2.7. Statistical Analysis

All numerical data are presented as mean ± standard deviation. All results were subjected to statistical analysis using a one-way ANOVA for multiple comparison of different samples. The significance level was set at *p* < 0.05.

## 3. Results and Discussion

### 3.1. Morphological Analysis

**Figure 1:**
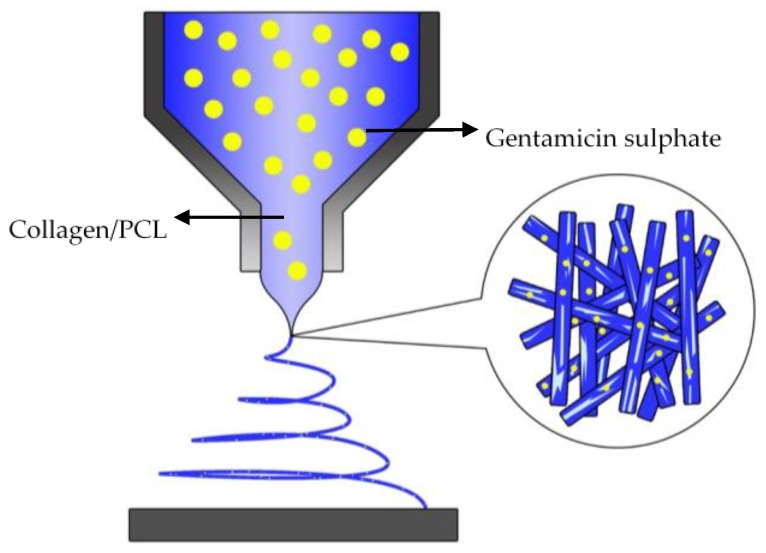
Illustration scheme of electrospun collagen/PCL loaded gentamicin sulfate at 12 kV and flow rate, 0.6 mL/h.

A collagen source from bovines is a natural polymer with bio-recognized physical and chemical properties. The complex structure of collagens, arranged as a triple helix singularly composed of amino acid sequences, may be easily compromised by the invasiveness of processing techniques commonly used to fabricate scaffolds [31]. However, the functionalization of scaffold via collagen macromolecules may represent an efficacious strategy to influence cellular interaction in order to mimic ECM functions [32]. Herein, the fabrication of PCL nanofibers embedded with collagen and drug (see Figure 1) has been proposed to develop instructive scaffolds for the local treatment of skin infections. As we know, PCL is a hydrophobic, and has a low degradation rate and a poor cell adhesion [33]. The addition of collagen with PCL nanofibers can improves the mechanical integrity of the matrix and thus, enhancing the cell adhesion and proliferation [34]. Moreover, it is expected with the addition of a hydrophilic phases might improve the hydrophobic response of PCL, thus addressing towards a better recognition of the nanofiber surface by cells in agreement with previous studies [35].

**Figure 2:**
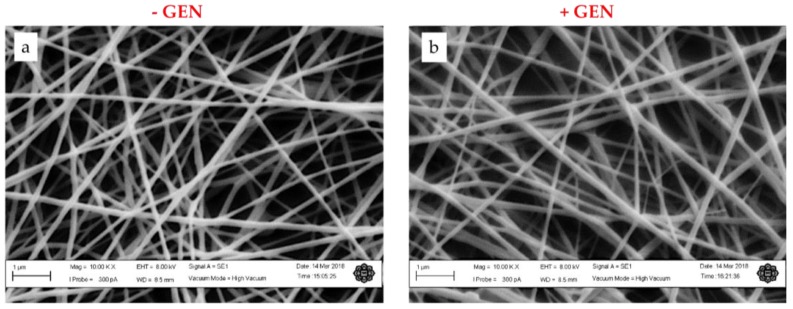
SEM images and fiber diameter distribution of (**a**) CP and (**b**) GCP electrospun fibers.

**Figure 3:**
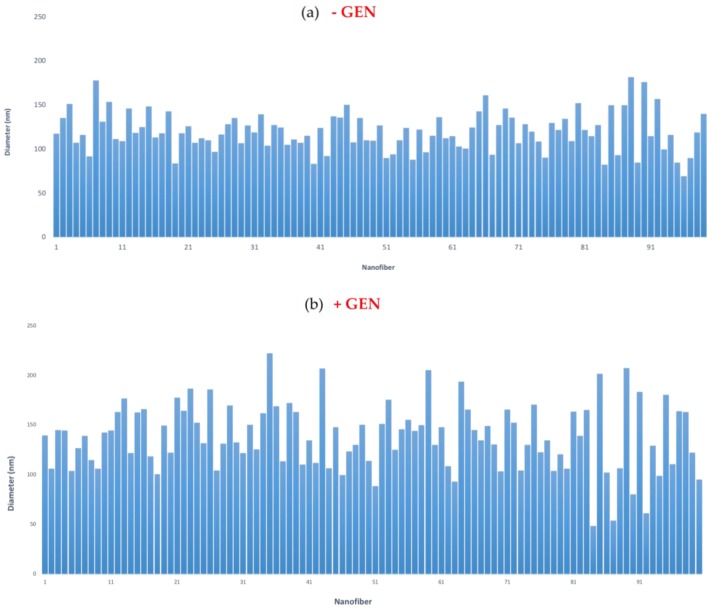
Fiber diameter distribution of (**a**) CP and (**b**) GCP electrospun fibers.

In this study, gentamicin was added in the collagen phase during the nanofibers preparation. By tuning the electrospinning parameters such as concentration, voltage, and flow rate, CP nanofibers were obtained. CP and GCP nanofibers were compared in terms of morphology and average fiber diameters. Scanning electron microscopy images (Figure 2a) revealed that CP nanofibers formed a randomly organized network with an average fiber diameter equal to 119.90 ± 21.97 nm (Figure 3a). In this case, a narrow distribution of fiber diameters was detected. This may be attributed to the chemical interactions between collagen and PCL, due to the formation of H-bonded intermolecular bonds among polymer entanglements [4], which favor a high fiber stretching during the electrospinning process. The incorporation of 3% gentamicin into collagen/PCL (Figure 2b) did not affect the relevancy of the morphology of fibers with an average diameter equal to 138.21 ± 33.41 nm (Figure 3b). It is noticed that GCP fiber diameter distribution was broader as compared to that of the CP, probably ascribable to the noisy contribution of drug molecules inserted among polymer chains during the fiber formation.

### 3.2. In Vitro Drug Release

**Figure 4:**
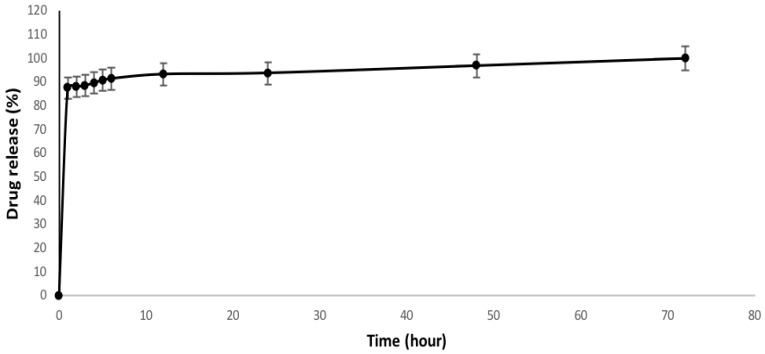
Release profile of GCP electrospun fibers.

Drug encapsulation efficiency of 81.1% was calculated, mainly ascribable to the good affinity of gentamicin with hydrophilic collagen, where the drug is mainly confined in nanofibers. The drug release profile of GCP is presented in Figure 4. A release of about 87.5% of the gentamicin is detected during the first 24 h with an initial burst release effect. The gentamicin tends to release at a slower rate until 72 h. In general, drug release depends on different mechanisms including diffusion, dissolution, drug desorption, and/or polymer degradation/erosion [36]. It is well-known that gentamicin shows low solubility with non-polar solvents—i.e., chloroform and methanol—but is highly soluble in polar ones like water. Hence, the initial burst release is the results of a rapid release of surface associated with drug molecules [19,37,38,39,40]. Moreover, we suggest that the mechanism of drug release is also influenced by the collagen dissolution mainly driven by water diffusion into a polymer network. Hence, the slow release of gentamicin from the nanofibers occurring after the burst release may be explained.

An extended initial burst release is required for a controlled delivery of antimicrobial drugs since it can support the fighting activity against intruding bacteria as cells begin to proliferate into the scaffold. A prolonged release of antimicrobial drug is also necessary to prevent further population.

The proposed release mechanism based on slow protein depletion properties is investigated in similar systems. Karuppuswamy et al. [40] used PCL membranes to control the release of tetracycline hydrochloride and showed high initial burst release, followed by a slow and steady release for 192 h. Due to the hydrophobic nature of PCL, slower diffusion of drugs is detected as the drug concentration increased. In other studies, Monteiro et al. [29] had investigated gentamicin-loaded liposome immobilized at the surface of chitosan nanofiber mesh to promote its antibacterial activity. Accordingly, gntamicin was successfully encapsulated into liposomes with an efficiency of 17%. gentamicin-loaded liposomes were uniformly distributed at the surface of the chitosan nanofiber mesh and the drug release kinetic showed a sustained release of gentamicin during first 16 h. In this case, the in vitro susceptibility tests confirmed that gentamicin released from the liposomes immobilized at the surface of electrospun chitosan nanofiber mesh has bactericidal activity against *Escherichia coli*, *Pseudomonas aeruginosa*, and *Staphylococcus aureus*. Moreover, in vivo study showed a wound treated with gentamicin-loaded patch revealed remarkably less scar at the closure of wounds, with an increase in size of collagen fibers and negligible inflammatory collection, so confirming a great potential for wound healing.

According to the drug release profile (Figure 4), GCP nanofibers confirmed to be an efficient drug’s reservoir with a controlled release profile, suitable to achieve and maintain optimum therapeutic drug levels in the blood as required for wound dressing applications.

### 3.3. In Vitro Cytotoxicity Test

**Figure 5:**
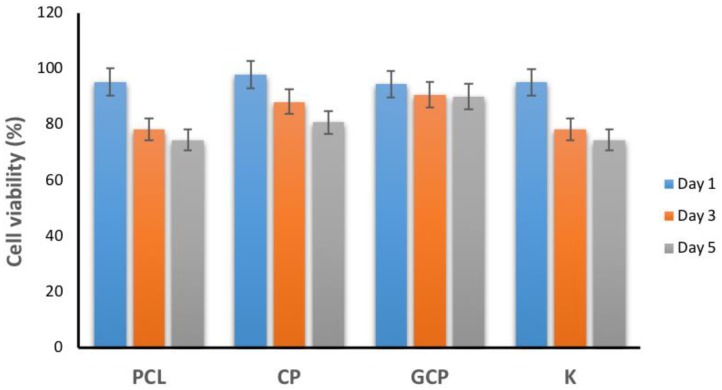
Cytotoxicity test by MTT assay on HDF cell culture onto PCL, CP, GCP nanofibers and K (culture plate) at day 1, 3, and 5.

The biocompatibility of PCL, CP, and GCP nanofibers with HDF after 1, 3, and 5 days were investigated using MTT assay (Figure 5) as the preliminary stage in their validation for clinical use. It is evaluated up to five days to see the validity of using collagen in vitro HDF activity. All samples exhibited no cytotoxicity up to five days. This result confirmed that CP and GCP nanofibers can promote cell growth and attachment, thus exhibiting no cytotoxicity for HDF cells. It can be assumed that both nanofibers are considered non-toxic towards the cell line as it displayed as no difference in sensitivity was observed between the nanofibers and control (K). Furthermore, GCP nanofibers can be used as a candidate for skin tissue regeneration.

## 4. Conclusions

Biodegradable collagen/PCL nanofibers containing hydrophilic gentamicin drug (an antibiotic drug) were fabricated by electrospinning and obtained fine nanoscale fibers diameter approximately 130 nm. The drug released from the nanofibers scaffold based on the drug erosion mechanism shows good controlled release up to 72 h. In vitro cytotoxicity response on human dermal fibroblast indicates no significant effect of nanofibers. In summary, collagen/PCL nanofibers have a good potential in skin tissue regeneration and drug delivery. Further studies in pre-clinical in vivo performance of nanofibers with regard to wound healing and tissue regeneration is encouraged.
